# Visual impairment in any eye adversely affects quality of life: Psychometric validation of the Malay NEI VFQ-25

**DOI:** 10.1371/journal.pone.0324979

**Published:** 2025-06-20

**Authors:** Jee Yao Loke, Sanjay Rampal, Jemaima Che Hamzah, Yi Wen Lim, Tengku Ain Kamalden

**Affiliations:** 1 Universiti Malaya Eye Research Centre, Department of Ophthalmology, Faculty of Medicine, Universiti Malaya, Kuala Lumpur, Malaysia; 2 Centre for Epidemiology and Evidence-Based Practice, Department of Social and Preventive Medicine, Faculty of Medicine, Universiti Malaya, Kuala Lumpur, Malaysia; 3 Department of Ophthalmology, Faculty of Medicine, Universiti Kebangsaan Malaysia, Kuala Lumpur, Malaysia; Universiti Malaya Fakulti Perubatan: University of Malaya Faculty of Medicine, MALAYSIA

## Abstract

**Purpose:**

To cross-culturally translate and adapt the National Eye Institute Visual Functioning Questionnaire (NEIVFQ-25) into Malay or Bahasa Malaysia, and to analyze its psychometric properties in a cohort of Malaysian patients with visual impairment from various causes.

**Design:**

Cross-sectional validation study

**Methods:**

The NEI-VFQ 25 was translated and cross-culturally adapted into the Malay version. Total of 324 visually impaired patients caused by cataracts, glaucoma, age-related macular degeneration (ARMD) or diabetic macular edema (DME), and a control group were included. Psychometric analysis was performed including test-retest and internal consistency reliability, convergent validity, discriminant validity, and factor analysis. Clinical validity was measured by correlation of clinical measurements with subdomain scores and known-groups comparison.

**Results:**

Participants’ average age was 60.4 years (SD 15.4) and 47.2% were male. The internal consistency was high in most subdomains, with Cronbach alpha ranging from 0.66–0.89. The test-retest reliability was high (intraclass correlation coefficient 0.92). Even when just one eye had impaired vision, participants scored much lower on the Malay NEIVFQ 25, highlighting how well the questionnaire reflects the real impact of visual impairment. Moderate correlations were detected between visual acuity and the ‘General vision’, ‘Near Activities’, ‘Distance Activities’, ‘Social Functioning’, ‘Mental Health’, ‘Role Difficulties’, ‘Dependency’ and ‘Driving’ subdomains suggesting that these subdomains were associated with central vision. Factor analysis demonstrated that 4 factors can be extracted from 12 subdomains.

**Conclusions:**

This study revealed that the Malay version of the NEIVFQ-25 is a valid, reliable, and reproducible instrument to measure the vision-related quality of life in patients with visual impairment. Quality of life was adversely affected even with only one eye having poor vision.

## Introduction

For the past decades, the scope of endpoints for evaluating medical treatments’ effect has widened and improved tremendously. There is a disparity between patients’ perception of treatment benefit and objective examination because these objective measurements do not evaluate the patients’ perceptions of their disease [1]. Nowadays, doctor-based evaluation of the burden of ophthalmic diseases is insufficient [2]. The objective clinical tests of visual acuity or visual field are inadequate to assess patients’ perception of visual impairment or vision-related to everyday living.

Health-related quality of life (HRQOL) is defined as health status in the physical, mental, and social domains, and the effect of a disease, its symptoms, and treatments on patients’ lives [3]. The conventional ophthalmic clinical examinations do not fully represent the impact of visual disability on the patients’ daily living activities. The impact of ophthalmic diseases on Quality of Life (QOL) has been demonstrated by Jacob et al [4]. Thus, we can have a broader view of the effect of ocular disease and its treatment on a patient’s life by measuring the vision-specific quality of life. However, in the field of ophthalmology, only a few instruments are available to measure Vision-related Quality of Life (VRQOL), such as the Quality of Life and Vision Function Questionnaire (QLVFQ) and Impact of Vision Impairment (IVI) [5,6].

One of the instruments, the 51-item National Eye Institute Visual Functioning Questionnaire (NEIVFQ-51), was developed to assess vision-related QOL [7]. It was later refined and shortened to a 25‑item version (the NEIVFQ‑25) to encourage higher response rates and improve the overall quality of the data [8]. The NEIVFQ −25 has 25 items that measure vision-targeted HRQOL and are grouped into 12 subdomains: general health (GH, one item); general vision (GV, one item); ocular pain (OP, two items); difficulty with near-vision activities (NV, three items); difficulty with distance-vision activities (DV, three items); limitation of social functioning because of vision (SF, two items); mental health problems because of vision (MH, four items); role limitations because of vision (RL, two items); dependency on others because of vision (DP, three items); driving difficulties (DR, two items); difficulty with color vision (CV, one item); and difficulty with peripheral vision (PV, one item). The questionnaire comprises 14 additional optional questions that can be used to substitute the items from each subdomain. Each subdomain score is converted to a score between 0 and 100 in which higher scores correspond to better vision-specific HRQOL. Except for the ‘General Health’ item, the composite VFQ-25 score is the mean score of all other items [7]. It has been translated and validated into many different languages such as Italian, Spanish, Turkish, Chinese, French, Japanese, Greece and has been widely utilized worldwide for clinical trials in many countries with different populations to describe the VRQOL of patients [3,9–15].

This study’s objective was to translate and adapt the NEIVFQ-25 into Malay cross-culturally and determine its psychometric properties.

## Methods

### Cross-cultural translation and adaptation of the Malay version of NEIVFQ-25

The English version of NEIVFQ-25 is available cost-free for all researchers at the website https://www.rand.org/health/surveys_tools/vfq.html [8]. It does not require permission to translate. The Malay version of NEIVFQ-25 was translated following international standard methods, including the forward translation, synthesis of translation, back translation, consultation by the expert committee, and pilot test.

The forward translation was carried out separately by two independent bilingual translators having Malay as mother tongue and more than 20 years of teaching and translational experience in the Linguistic Faculty of the University of Malaya. Neither of them had prior knowledge of the NEIVFQ-25. Upon completing the two independent translations, both translation copies from the 2 independent translators above were compared, and the discrepancies were identified. There were no difficulties encountered when translating from the English version to Malay version. Following an expert panel meeting, an agreed version of Malay version NEIVFQ-25 was conceived.

The agreed-upon Malay version NEIVFQ-25 was then translated into English by another independent translator who had never seen the original English version of the questionnaire. It was performed by a qualified bilingual translator whose primary language is English. The difficulties encountered in the course of the translation were identified.

Discussion between the translators and clinical specialists identified significant conceptual and semantic differences reaching the original questionnaire with special attention given to any difficulty understanding the questions. The committee discussed the discrepancies indirectly translated versions, the summarized version, and then the back-translated version.

A pilot test was performed on ten patients with visual impairment recruited from the University of Malaya Medical Centre (UMMC) Ophthalmology Clinic to assess the clarity, cultural relevance, and adaptation of the Malay version NEIVFQ-25 to Malay patients’ experience. A minimal revision was performed to finalize the Malay version of NEIVFQ-25 used in this study.

### Psychometric evaluation study

Recruitment began on the 15^th^ July 2019 and ended on the 15^th^ Sept 2019. Written informed consent was obtained from each participant. Assessment of the reliability and validity of the Malay version of NEIVFQ-25 was performed on 324 participants who visited UMMC Ophthalmology clinic with the following inclusion criteria: (i) must be 21 years of age or older and understand the Malay language; (ii) no psychiatric illness or intellectual disability; (iii) clinical evidence of age-related cataracts, glaucoma, age-related macular degeneration (ARMD), diabetic macular edema(DME) or regular patient with corrected refractive error. We selected a minimum age of 21 years based on several key considerations. Persons aged 21 years is widely recognized as the age of full legal adulthood, which ensures that participants can provide informed consent independently. Moreover, individuals at this age are typically considered to have the necessary cognitive maturity and life experience to reliably assess their vision-related quality of life. This age threshold is also consistent with prior validation studies of the NEI VFQ-25 that have focused on adult populations [7,8], thereby enhancing the comparability and reliability of our findings.

Visual acuity scores were recorded in Snellen visual acuity and converted to LogMAR before analysis using the formula ‘LogMAR = -log10(decimal acuity)’.

For patients with cataracts, the inclusion criteria had cataracts in both eyes and Snellen visual acuity of 6/12 or worse in the worse eye. Inclusion criteria for glaucoma patients were primary or secondary glaucoma, with at least once documented abnormality in a Humphrey visual field analyzer or optic nerve defect and no ocular surgery for glaucoma treatment during the previous three months. Patients with ARMD must have clinical signs of macular degeneration with optical coherence tomography (OCT) evidence. For patients with DME, the inclusion criteria were at least 6/12 or worse Snellen visual acuity in the one eye with OCT evidence of center-involving macular edema. One reference group of regular participants with corrected refractive error.

Cross-sectional study design was used, and the Malay version NEIVFQ-25 was administered to all 324 participants. A total of 47 participants were randomly chosen by convenience sampling to repeat the questionnaire a second time with 1–2 weeks apart for the evaluation of test-retest reliability. The duration was set for 1–2 weeks apart as this time frame was estimated to be adequate time for patients to forget their initial answers, but short enough to avoid longitudinal changes in visual acuity.

The sociodemographic factors measured were gender, age, ethnicity, marital status, occupational status, and educational level. In the Malaysian education system, children aged 7–12 years attend primary school, those aged 13–17 years attend secondary school, and tertiary education covers post-secondary programs for students 18 years and above, including pre-university courses and undergraduate degrees. Clinical data included visual acuity and the number of comorbidities.

All questionnaires were administered by three trained interviewers not involving in the medical care of the patients. The UMMC-Medical Research Ethics Committee approved this study of (Ref No.201928−7114). Written informed consent was obtained from all participants. This study was carried out in accordance with the Declaration of Helsinki.

### Statistical analysis

All analyses were carried out in SPSS version 25, using p-value to determine statistical significance. The specific methods used are detailed in the following sections.

### Descriptive analysis and item analysis

Categorical data were summarized using frequency and proportions [n (%)]. Continuous data were summarized as means and standard deviation (mean±SD) when normally distributed. For skewed distributions, the distributions were summarized as medians and interquartile ranges. The number of non-response items (missing values) and the percentage of item response at the ceiling (highest possible score) and floor (lowest possible score) of the Malay NEIVFQ-25 was examined.

### Reliability

Cross-sectional data from the samples were used to analyse reliability. Internal consistency is quantified with the Cronbach alpha coefficient, which is a measure of the extent to which items within a single subdomain correlate with each other [16]. The optimal range of Cronbach alpha is 0.70–0.90. The Intraclass Correlation Coefficient (ICC) were calculated to evaluate test-retest reliability [17]. A reliability index of above 0.70 is considered satisfactory.

### Validity

The multi-trait analysis was used to evaluate convergent and discriminant validity [18]. Each item is hypothesized to belong to only one multi-item subdomain:

1. We computed the correlations between the score of each item and the scores on all the subdomains.
2. For each item, if the correlation between the item and subdomain scores (to which the item belongs) is 0.4 or higher, that item is deemed to have “passed” the test of convergent validity.
3. If the correlation between the item and subdomain scores (to which the item belongs) is significantly greater than the correlations between the item and the total subdomain scores (to which the item does not belong), then that item is deemed to have “passed” the test of discriminant validity.

For each subdomain, the convergent validity was computed as the percentage of its items with a corrected item-scale correlation of at least 0.4. The item discriminant validity was stated as the percentage of items whose corrected item-domain correlation was greater than the correlation with other domains [18].

Clinical validity was determined by comparing the Malay NEIVFQ-25 scores from the participants with poorer vision with good vision. We analysed the subdomain scores across different visual acuity (LogMAR) of the better eye and worse eye. The composite scores and individual subdomain component scores across different ocular conditions and visual acuity were calculated and analyzed with the Kruskal-Wallis test and Mann-Whitney test. We also computed the Spearman correlation between all subdomain scores with visual acuity and visual field scores of the better eye and worse eye.

Lastly, factor analysis was used to determine the one-dimensionality of the scale. This was performed with the principal axis factoring method and varimax rotation.

## Results

### Translation and pilot test

The pilot-testing results demonstrated that the patients well accepted the Malay NEIVFQ-25 as the test duration was short, and all items were easy to understand. After discussion among the expert panel, a slight modification of one question was performed to adapt the questionnaire to Malay patients’ experience properly. Therefore, the optional question in ‘A7’—which originally mentioned golf, bowling, jogging, or walking—was revised by replacing ‘bowling’ with ‘badminton’, a sport more popular in Malaysia and the South East Asia region.

### Psychometric evaluation

There was a total of 324 participants included in the study. Seventy-four participants repeated the questionnaire after 1–2 weeks. The participants consisted of 153 males (47.2%) and 171 females (52.8%). The mean age was 60.4 ± 15.4 years. The majority of the participants were Malay (n = 226, 69.8%). The participants’ characteristics are shown in Table 1.

**Table 1:** Demographics and clinical characteristics of study participants. Data are presented as mean (±SD) for continuous outcomes.

	n (%)
Gender	
Male	153 (47.2)
Female	171 (52.8)
Age (years)	
21-40	49 (15.1)
41-60	77 (23.8)
>60	198 (61.1)
Mean of age	60.4 ± 15.4 years
Ethnicity	
Malay	226 (69.8)
Indian	47 (14.5)
Chinese	46 (14.2)
Others	5 (1.5)
Marital status	
Single	40 (12.3)
Married	277 (85.5)
Widower	7 (2.2)
Educational status^#^	
Illiterate	3 (0.9)
Primary	57 (17.6)
Secondary	144 (44.4)
Tertiary	120 (37.0)
Working status	
Working	76 (23.5)
Unemployed	248 (76.5)
No. of systemic diseases	
None	75 (23.1)
1	83 (25.6)
2	119 (36.7)
3	43 (13.3)
≥4	4 (1.2)
Principle diagnosis	
Cataract	116 (35.8)
Glaucoma	77 (23.8)
DME	67 (20.7)
ARMD	21 (6.5)
None	43 (13.3)
LogMAR VA (better-seeing eye)	
0 - 0.59	265 (81.8)
0.6 - 1.29	51 (15.7)
>1.3	8 (2.5)
LogMAR VA (worse-seeing eye)	
0 - 0.59	165 (50.9)
0.6 −1.29	85 (26.2)
>1.3	74 (22.8)

MAR, minimum angle of resolution; VA, visual acuity.

A total of 5 groups of participants with different principle diagnoses were studied, namely cataract (n = 116,35.8%), glaucoma (n = 77,23.8%), ARMD (n = 21,6.5%), DME(n = 67,20.7%) and control group with no ocular pathologies (n = 43, 13.3%). Majority of the participants had visual acuity of better than LogMAR 0.6 (81.8%) in better-seeing eye. (Table 1)

The number and percentages of missing responses, floor responses (the lowest score), and ceiling responses (the highest score) for each item are shown in Table 2. ‘Not doing the suggested activity for reasons other than visual problem’ were recorded as missing responses for each question. ‘Going out to movies or plays’ from the ‘Distant Activities’ subdomain was not endorsed by 52.5% of the participants, while ‘Participating in sports or outdoors’ was included in the additional question for the “Distant Activities’ subdomain, was not endorsed by 36.4% of respondents. Furthermore, higher than 35% of missing responses for the three questions in the ‘Driving’ subdomain. The floor effect was not seen in any of the subdomains. On the other hand, the percentage of participants scoring at the ceiling was over 20% in 10 out of 12 subdomains (ocular pain, near vision, distance vision, social functioning, mental health, role limitation, dependency, driving, color vision, and peripheral vision) (Table 2).

**Table 2:** Item analysis on missingness, floor and ceiling responses (n = 324).

Subdomain and Item	Question number	Missing responses n(%)	Floor responses n(%)	Ceiling responses n(%)
**General health:**				
5-level health rating	1	0	14 (4.3)	20 (6.2)
**General vision:**				
6-level general vision	2	0	1 (0.3)	19 (5.9)
**Ocular pain:**				
Amount of pain	4	0	1 (0.3)	204 (63.0)
Amount of time: pain	19	0	4 (1.2)	220 (67.9)
**Near activities:**				
Reading normal newsprint	5	9 (2.8)	45 (13.9)	118 (36.4)
See well up close	6	33 (10.2)	34 (10.5)	165 (50.9)
Finding objects on a crowded shelf	7	7 (2.2)	5 (1.5)	221 (68.2)
**Distance activities:**				
Reading street signs	8	1 (0.3)	9 (2.8)	166 (51.2)
Going down stairs at night	9	12 (3.7)	17 (5.2)	150 (46.3)
Going out to movies/plays	14	170 (52.5)	10 (3.1)	112 (34.6)
**Social functioning:**				
Seeing how people react	11	1 (0.3)	1 (0.3)	257 (79.3)
Visiting others	13	13 (4.0)	12 (3.7)	226 (69.8)
**Mental health:**				
Amount true: worry	3	0	51 (15.7)	79 (24.4)
Amount true: frustrated	21	0	46 (14.2)	213 (65.7)
Amount true: no control	22	1 (0.3)	20 (6.2)	243 (75.0)
Amount true: embarrassment	25	0	15 (4.6)	260 (80.2)
**Role limitations:**				
Accomplish less	17	0	21 (6.5)	198 (61.1)
Limited in endurance	18	0	53 (16.4)	175 (54.0)
**Dependency:**				
Stay home most of the time	20	0	44 (13.6)	223 (68.8)
Rely too much on other’s word	23	0	37 (11.4)	197 (60.8)
Need much help from others	24	0	41 (12.7)	200 (61.7)
**Driving:**				
Daylight familiar places	15c	114 (35.2)	47 (14.5)	128 (39.5)
Driving at night	16	161 (49.7)	27 (8.3)	55 (17.0)
Driving in difficult conditions	16a	163 (50.3)	23 (7.1)	70 (21.6)
**Colour vision:**				
Difficulty matching clothes	12	6 (1.9)	1 (0.3)	293 (90.4)
**Peripheral vision:**				
Seeing objects off to the side	10	0	1 (0.3)	218 (67.3)
**OPTIONAL ITEMS**				
**General Health:**				
11-level health rating	A1	0	1 (0.3)	24 (7.4)
**General Vision:**				
11-level health rating	A2	0	2 (0.6)	21 (6.5)
**Near activities:**				
Reading small print	A3	4 (1.2)	45 (13.9)	131 (40.4)
Reading mail/bills accurately	A4	6 (1.9)	42 (13.0)	181 (55.9)
Shaving/styling hair/makeup	A5	57 (17.6)	8 (2.5)	230 (71.0)
**Distance activities:**				
Recognizing faces in room	A6	1 (0.3)	8 (2.5)	181 (55.9)
Participating in sports/outdoors	A7	118 (36.4)	17 (5.2)	157 (48.5)
Seeing television program	A8	11 (3.4)	10 (3.1)	202 (62.3)
**Social functioning:**				
Entertaining friends	A9	9 (2.8)	2 (0.6)	282 (87.0)
**Role limitations:**				
More help from others	A11a	0	20 (6.2)	207 (63.9)
Limited in kinds of things that can do	A11b	0	41 (12.7)	190 (58.6)
**Mental health:**				
Irritable	A12	0	8 (2.5)	255 (78.7)
**Dependency:**				
Don’t go out alone	A13	0	43 (13.3)	221 (68.2)

The Cronbach Alpha coefficient, which reflects the internal consistency, was higher than 0.70 for almost all subdomains, as shown in Table 3. Cronbach Alpha coefficient was lower only in ‘Social Functioning’ and ‘Driving’ subdomains at 0.655 & 0.659, respectively. Regarding test-retest reliability, the ICC was 0.70 or higher in all subdomains except ‘General Health,’ ‘Ocular Pain,’ and ‘Color Vision’ subdomains (Table 3). All items passed the convergent validity test, and closed to 100% of items passed discriminant validity tests.

**Table 3:** Reliability and validity analysis.

Subdomain	Number of Items	Cronbach Alpha	Intraclass Correlation Coefficients (Test-Retest Reliability)	Convergent Validity^*^	Discriminant Validity^**^
General Health	2	0.795	0.670	100	100
General Vision	2	0.849	0.731	100	100
Ocular Pain	2	0.707	0.433	100	100
Near Activities	6	0.888	0.875	100	100
Distance Activities	6	0.840	0.895	100	100
Social Functioning	3	0.655	0.810	100	100
Mental Health	5	0.758	0.835	100	100
Role Difficulties	4	0.829	0.819	100	97.7
Dependency	4	0.885	0.865	100	100
Driving	3	0.659	0.940	100	100
Colour Vision	1	NA	NA	NA	NA
Peripheral Vision	1	NA	0.747	NA	NA
Composite Score	39	0.922	0.917	NA	NA

* The percentage of items that passed the test of convergent validity.

** The percentage of items that passed the test of discriminant validity.

NA : Not applicable to single-item scales.

The composite and subdomain scores for different ocular conditions are presented in Table 4. The composite NEI-VFQ median scores for the control group (97.3) was significantly higher than the cataract (79.2), glaucoma (85.6), ARMD (77.9), and DME (78.5) groups, respectively. The median of the control group was significantly higher for all subdomain scores except for color vision. The composite scores across different visual acuity (Log MAR) of the better eye and worse eye are illustrated in Table 5. It showed that as the participants’ visual acuity deteriorates (higher LogMAR value), the composite scores dropped.

**Table 4:** Discriminant validity of composite and subdomain scores by various ocular conditions.

	Diseased				Normal (N = 43)	P-value*
NEIVFQ-25 subdomain	Cataract (N = 116)	Glaucoma (N = 77)	ARMD (N = 21)	DME (N = 67)		
	Median(Q1,Q3)	Median(Q1,Q3)	Median(Q1,Q3)	Median(Q1,Q3)	Median(Q1,Q3)	
**Composite score**	**79.2** (62.2,90.9)	**85.6** (75.4,93.2)	**77.9** (71.5,83.8)	**78.5** (64.7,87.9)	**97.3** (94.8,98.6)	<0.001
**Subdomains score**						
**General Health**	55.0 (42.5,65.0)	55.0 (45.0,65.0)	60.0 (51.3,65.0)	47.5 (37.5,60.0)	82.5 (65.0,100)	<0.001
**General Vision**	55.0 (45.0,65.0)	60.0 (50.0,70.0)	55.0 (52.5,60.0)	55.0 (45.0,70.0)	85.0 (80.0,100)	<0.001
**Ocular Pain**	100 (75.0,100)	87.5 (62.5,100)	100 (100,100)	87.5 (75.0,100)	100 (100,100)	<0.001
**Near Activities**	75.0 (46.9,87.5)	91.7 (79.2,100)	75.0 (56.3,91.7)	79.2 (54.2,87.5)	100 (100,100)	<0.001
**Distance Activities**	80.0 (55.3,95.0)	87.5 (75.0,100)	83.3 (66.7,94.4)	83.3 (62.5,95.0)	100 (91.7,100)	<0.001
**Social Functioning**	95.8 (77.1,100)	100 (91.7,100)	100 (85.4,100)	100 (91.7,100)	100 (100,100)	<0.001
**Mental Health**	80.0 (56.3,95.0)	90.0 (70.0,100)	80.0 (67.5,90.0)	75.0 (55.0,90.0)	95.0 (90.0,100)	<0.001
**Role Difficulties**	75.0 (50.0,100)	87.5 (62.5,100)	68.8 (50.0,81.3)	68.8 (50.0,93.8)	100 (100,100)	<0.001
**Dependency**	81.3 (45.3,100)	100 (65.6,100)	81.3 (62.5,84.4)	75.5 (37.5,100)	100 (100,100)	<0.001
**Driving**	58.3 (16.7,83.3)	75.0 (0,89.6)	0 (0, 58.3)	66.7 (25.0,83.3)	100 (83.3,100)	<0.001
**Colour Vision**	100 (100,100)	100 (100,100)	100 (100,100)	100 (100,100)	100 (100,100)	0.609
**Peripheral Vision**	100 (75.0,100)	100 (62.5,100)	100 (75.0,100)	100 (75.0,100)	100 (100,100)	<0.05

* Analysed using Kruskal-Wallis test.

**Table 5:** Discriminant validity of composite scores stratified by combination of better and worse eye for those with abnormal ocular conditions (n = 281).

Composite Score Median(Q1, Q3)		Worse Eye			P-value *
	Log MAR	0 - 0.59	0.6–1.29	>1.3	
**Better Eye**	**0 - 0.59**	**86.4** (77.7,93.0) (n = 123)	**79.9** (64.7,91.6) (n = 59)	**78.8** (60.9,87.8) (n = 40)	**<0.001**
	**0.6–1.29**	N/A	**74.1** (58.4,86.3) (n = 25)	**67.9** (45.3,80.4) (n = 26)	
	**>1.3**	N/A	N/A	**40.7** (26.6,56.3) (n = 8)	

* Analysed using Kruskal-Wallis test.

There was a negative and moderate correlation between LogMAR and the composite score; these correlations were similar when using LogMAR of the better eyes compared to the worse eyes (Table 6). Visual acuity scores positively correlated with subdomains of central vision: General Vision, Near Activities, Distance Activities, Social Functioning, Mental Health, Role Difficulties, Dependency, and Driving (P < 0.05). Visual field scores were moderately negatively correlated with composite score and most of the subdomain scores except General Health and Ocular Pain (P < 0.05).

**Table 6:** Correlation of Malay NEI-VFQ 25 composite and subdomain scores with visual functions.

	LogMAR, ρ		Visual Field, ρ	
NEIVFQ-25 subdomains	*Better Eye*	*Worse Eye*	*Better eye*	*Worse eye*
**Composite score**	**−0.55** ^*^	**−0.59** ^*^	**0.66** ^*^	**0.67** ^*^
**General Health**	−0.31^*^	−0.30^*^	0.16	0.10
**General Vision**	**−0.51** ^*^	**−0.56** ^*^	**0.55** ^*^	**0.52** ^*^
**Ocular Pain**	−0.08	−0.20^*^	0.10	0.27^*^
**Near Activities**	**−0.58** ^*^	**−0.50** ^*^	0.30^*^	0.34^*^
**Distance Activities**	**−0.51** ^*^	**−0.46** ^*^	**0.56** ^*^	**0.48** ^*^
**Social Functioning**	**−0.47** ^*^	**−**0.39^*^	**0.52** ^*^	**0.60** ^*^
**Mental Health**	**−0.41** ^*^	−0.38^*^	**0.45** ^*^	**0.44** ^*^
**Role Difficulties**	**−0.45** ^*^	**−0.52** ^*^	**0.62** ^*^	**0.49** ^*^
**Dependency**	**−0.49** ^*^	**−0.48** ^*^	**0.54** ^*^	**0.56** ^*^
**Driving**	**−0.47** ^*^	**−0.57** ^*^	**0.68** ^*^	**0.59** ^*^
**Colour Vision**	−0.27^*^	−0.16^*^	0.36^*^	0.32^*^
**Peripheral Vision**	−0.26^*^	−0.33^*^	**0.52** ^*^	**0.56** ^*^

* Correlation was tested using Spearman’s ^ρ^. P < 0.05 indicates a two-sided significant correlation.

Interpretation of correlation: i) weak is ±0.10< ^ρ^<±0.39; ii) moderate is ±0.40< ^ρ^<±0.69; iii) strong is ^ρ^>0.7 or ^ρ^ < − 0.7.

Bold characters indicate correlation coefficients of 0.4 or greater.

MAR , minimum angle of resolution.

Visual field testing with the Humphrey Visual Field Analyzer (only glaucoma, n = 63).

Following the factor analysis of 12 NEI-VFQ subdomains, four factors were extracted that explained a cumulative variance of 77.4% (Table 7). Factor 1 explained 53.8% of the Variance and consisted of Visual related QOL of near activities, distance activities, social functioning, color vision, and peripheral vision. The remaining three factors explained 23.6% of the total Variance.

**Table 7:** Factor Analyses and its loadings of all subdomains of Malay NEIVFQ-25.

Subdomain	Factor 1	Factor 2	Factor 3	Factor 4
General Health			0.66	
General Vision			0.81	
Ocular Pain				0.56
Near Activities	0.56	0.58		
Distance Activities	0.65	0.50		
Social Functioning	0.84			
Mental Health		0.56		
Role Difficulties		0.71		
Dependency		0.77		
Driving			0.46	
Colour Vision	0.59			
Peripheral Vision	0.52			
% Variance Explained	53.8%	9.5%	8.0%	6.0%

Kaiser-Meyer-Olkin value – 0.901; Cumulative variance explained by 4 factors are 77.4%.

Factor loading value of less than 0. 4 is omitted.

## Discussion

We validate and cross-culturally adapt a Malay (for Malaysia) version of the NEI VFQ-25 and reported its detailed psychometric properties across various ocular conditions. Although a similar validation and translation study of the NEI VFQ into Malay has been reported [19], our work significantly extends these efforts. Unlike the study by Thevi et al. which focused solely on cataract patients, our research includes a diverse sample of 324 patients with various ocular conditions—including cataract, glaucoma, ARMD, and DME—as well as a control group, enhancing the generalizability of our findings. We implemented a rigorous translation process with independent forward and backward translations, expert panel review, and pilot testing to ensure cultural and conceptual equivalence. Moreover, we evaluated not only internal consistency but also test–retest reliability, convergent and discriminant validity, and clinical validity by correlating VFQ‑25 scores with objective measures such as LogMAR visual acuity and visual fields. Our advanced statistical analyses, which include a four‑factor model and detailed item response theory analysis, provide a deeper understanding of the instrument’s psychometric properties—addressing key limitations in their approach [19]. We found that this Malay version could provide reliable, valid, and responsive data to change in visual function.

The Malay NEIVFQ-25 questionnaire’s subdomains had high internal consistencies (>0.7), indicating this instrument’s high reliability in our population. Moreover, the short time window between test and retest should prevent any significant changes in the participants’ systemic or vision-related functional status. High Intraclass Correlation Coefficients of more than 0.7 in most subdomain scores indicate that Malay NEIVFQ-25 had strong test-retest reliability. On the other hand, the ICC value of the ‘Ocular Pain’ subdomain is below 0.5. It may be due to the participants’ subjective feelings or the fact that ocular pain may change within two weeks. Overall, Malay NEIVFQ-25 has high internal consistency and test-retest reliability. The result of these correlations is similar to those observed in NEI psychometric field test [8].

Research consistently shows that losing vision in one eye can lead to a lower quality of life, increased psychological distress, and challenges in social interactions [20,21]. It not only hampers physical abilities but also takes a toll on emotional, mental, and social health. Our results corroborates this finding: participants with poorer vision (higher LogMAR) were found to have reduced quality of life based on lower scores. The ability of the Malay NEIVFQ-25 to demonstrate significant changes in NEIVFQ scores (Table 5) with respect to different levels of VA loss indicates satisfactory clinical validity.

Lower scores were observed among study participants with ocular diseases compared to normal eyes (Table 4). On the other hand, no difference in ‘Colour Vision’ subdomain scores may be due to the relatively late color vision involvement among these ocular diseases. Similar findings were reported in previous studies [11,12].

Additionally, moderate correlations were detected between VA and the visual field of the eye of the participants and the ‘General vision,’ ‘Near Activities,’ ‘Distance Activities,’ ‘Social Functioning,’ ‘Mental Health,’ ‘Role Difficulties,’ ‘Dependency’ and ‘Driving’ subdomains suggesting that these subdomains were associated with central vision (Table 6). The other subdomains, ‘General Health,’ ‘Ocular Pain,’ ‘Colour Vision,’ and ‘Peripheral vision,’ correlated weakly with VA. Similar correlations between VA and NEI-VFQ subdomains had been identified previously during the validation of the NEIVFQ-25 in other languages [3,11,12].

Exploratory factor analysis revealed that four factors were extracted from the 12 subdomains (Table 7). Factor 1 are subdomains affiliated to visual acuity such as ‘Near Activities,’ ‘Distance Activities,’ ‘Social functioning,’ ‘Colour Vision,’ and ‘Peripheral vision.’ ‘Driving’ subdomain are clustered with ‘General Health,’ ‘General Vision,’ in factor 3. This suggests that driving is associated with good vision and general health, and vice versa. Lastly, the ‘Ocular Pain’ subdomain stood alone as a factor. This analysis implies that the Malay version of NEIVFQ-25 may be shortened to fewer questions.

## Study limitations

This study was conducted using convenient sampling of participants with common ocular conditions at a single tertiary centre in Malaysia. Extrapolation of these results to other types of ocular conditions or populations is unknown.

Secondly, high rates of missing data were observed for items ‘Going out to movies or plays,’ ‘Participating in sports or outdoors’ in the ‘Distance Activities’ subdomain, and all items in the ‘Driving’ subdomain (Table 2). Similar results were also observed during the translation and validation of the NEI-VFQ 25 in other populations [3,8,13]. In our population, because most of our study participants are in the older age group and are pensioners, their daily activities are likely to be far less active. Thus, it is highly possible that respondents may have skipped these questions as these activities may no longer be relevant to them. Suzukamo et al. substituted items with a high rate of missing data with those with low rates of missing data, on the provision that the percentage of ceiling or floor responses did not exceed 50% [3]. Thus, we suggest that in Malay NEIVFQ, the ‘Driving’ subdomain may be categorized as optional.

## Conclusion

In conclusion, our study confirms that this Malay NEI VFQ-25 version is a valid, reliable, and broadly applicable instrument for assessing vision-related quality of life across multiple ocular diseases. Notably, it captures how having poor vision in a single eye can lower the overall score, underscoring its sensitivity to unilateral visual impairment. Its robust psychometric properties and cultural relevance make it highly suitable for Malay-speaking populations, not only in Malaysia but also in other Southeast Asian countries—such as Singapore and Brunei—where Malay serves as a common lingua franca. This questionnaire is therefore an excellent validated tool for clinical trials, quality-of-life assessments, and health economic research in this region.

## Supporting information

S1 File Malay NEIVFQ_2025. (PDF)
