# Supplementary material for: Visual impairment in any eye adversely affects quality of life: Psychometric validation of the Malay NEI VFQ-25
Source: PLoS One. 2025 Jun 20;20(6):e0324979. doi: 10.1371/journal.pone.0324979 (PMC12180719; doi:10.1371/journal.pone.0324979)
Supplement: S1 File — (PDF) [file pone.0324979.s001.pdf]

Institut Mata Negara

Soal Selidik Fungsi Penglihatan - 25

(VFQ-25)

versi 2000

(FORMAT YANG DIKENDALIKAN OLEH PENEMUBUAL)

RAND dengan ini memberi keizinan untuk menggunakan “Soal Selidik Fungsi Penglihatan - 25 (VFQ-25), Julai 1996, Institut Mata Negara, mengikut syarat-syarat berikut yang hendaklah diandaikan telah dipersetujui oleh semua pihak sebagai akibat daripada penerimaan pakai dokumen ini:

1. Perubahan kepada NEI VFQ-25 – Julai 1996 boleh dilakukan tanpa kebenaran bertulis daripada RAND. Walau bagaimanapun, semua perubahan tersebut mestilah dikenalpasti dengan jelas sebagai sesuatu yang dilakukan oleh penerima.

2. Pengguna NEI VFQ -25 – Julai 1996 ini bertanggungjawab sepenuhnya , dan bersetuju untuk berpegang bahawa RAND tidak boleh dipersalahkan untuk ketepatan mana-mana terjemahan NEI VFQ -25 Versi Ujian – Julai 1996 ke dalam bahasa yang lain dan untuk mana-mana kesilapan, ketinggalan, salah tafsir ataupun akibat daripada perbuatan tersebut.

3. Pengguna NEI VFQ -25 – Julai 1996 ini bertanggungjawab sepenuhnya, , dan bersetuju untuk berpegang bahawa RAND tidak boleh dipersalahkan untuk sebarang akibat daripada penggunaan NEI VFQ -25.

4. Pengguna NEI VFQ -25 – Julai 1996 akan memberi sebaris penghargaan apabila mencetak dan mengedar dokumen ini ataupun dalam pencetakan keputusan atau analisis yang berdasarkan instrumen ini dengan mengakui bahawa ia dibentuk oleh RAND di bawah tajaan Institut Mata Negara.

5. Tiada sebarang kebenaran bertulis diperlukan untuk penggunaan NEI VFQ-25 - Julai 1996 ini.

7/29/96

© **RAND** 1996

Arahan:

Saya akan bacakan beberapa kenyataan tentang masalah yang melibatkan penglihatan atau perasaan anda tentang keadaan penglihatan anda. Selepas setiap soalan saya akan bacakan satu senarai jawapan yang mungkin benar. Sila pilih jawapan yang paling sesuai untuk menerangkan keadaan anda.

Sila jawab semua soalan seolah-olah anda sedang memakai cermin mata ataupun kanta lekap anda.

Sila ambil masa yang diperlukan untuk menjawab setiap soalan. Kesemua jawapan anda adalah sulit. Untuk memastikan soal selidik ini dapat meningkatkan pengetahuan kami tentang masalah penglihatan dan bagaimana ia memberi kesan kepada kualiti hidup anda, jawapan anda mestilah setepat mungkin. Adalah diingatkan, sekiranya anda memakai cermin mata atau kanta lekap untuk sesuatu aktiviti, sila jawab soalan-soalan berikut seolah-olah anda sedang memakainya.

Soal Selidik Fungsi Penglihatan - 25

BAHAGIAN 1 – KESIHATAN UMUM DAN PENGLIHATAN

1. **<u>Secara umum</u>, bolehkah dikatakan bahawa kesihatan anda secara keseluruhannya adalah * :**

(Bulatkan Satu)

BACAKAN KATEGORI:

Tersangat Baik 1
Sangat Baik 2
Baik...3
Sederhana 4
Lemah.......................................5

2. **Pada masa ini, bolehkah dikatakan penglihatan kedua-dua belah mata anda (sekiranya anda memakai samada cermin mata atau kanta lekap) adalah sangat baik, baik, sederhana, lemah, atau sangat lemah atau adakah anda buta sepenuhnya?**

(Bulatkan Satu)

BACAKAN KATEGORI:

Sangat Baik 1
Baik.. 2
Sederhana 3
Lemah...................................... 4
Sangat Lemah.......................... 5
Buta Sepenuhnya.................... 6

*** Langkau Soalan 1 apabila VFQ-25 dijalankan pada masa yang sama dengan SF-36 atau RAND 36-Item Health Survey 1.0**

3. **Selalukah anda <u>merasa bimbang</u> tentang penglihatan anda?**

(Bulatkan Satu)

BACAKAN KATEGORI:

Tidak langsung 1
Jarang-jarang …….......................... 2
Kadang-kadang 3
Selalu.. 4
Setiap masa?.................................... 5

4. **Apakah tahap <u>kesakitan atau ketidakselesaan</u> yang anda hadapi <u>di dalam</u> atau <u>di sekeliling</u> mata (seperti rasa panas, gatal ataupun sakit)? Adakah ianya:**

(Bulatkan Satu)

BACAKAN KATEGORI:

Tiada.. 1
Sedikit 2
Sederhana.. 3
Sakit 4
Sangat sakit....................................... 5

BAHAGIAN 2 – KESUKARAN MELAKUKAN AKTIVITI

Soalan berikutnya adalah tentang kesukaran yang mungkin anda hadapi untuk melakukan aktiviti dengan memakai cermin mata atau kanta lekap semasa melakukan aktiviti tersebut.

5. **Adakah sukar bagi anda untuk <u>membaca cetakan biasa di dalam surat khabar</u>?**
   (BACAKAN KATEGORI YANG DIPERLUKAN)

(Bulatkan Satu)

Tidak sukar langsung 1
Sedikit sukar.. 2
Sederhana sukar.. 3
Sangat sukar... 4
Berhenti melakukannya kerana masalah penglihatan 5
Berhenti melakukannya kerana sebab-sebab lain atau tidak berminat untuk melakukannya 6

6. **Adakah sukar bagi anda untuk melakukan kerja atau hobi yang memerlukan anda melihat dengan baik pada jarak yang dekat, seperti memasak, menjahit, membaiki barang-barang di rumah, atau menggunakan alatan tangan? Adakah anda rasakan:**
(BACAKAN KATEGORI YANG DIPERLUKAN)

(Bulatkan Satu)

Tidak sukar langsung 1
Sedikit sukar.. 2
Sederhana sukar.. 3
Sangat sukar.. 4
Berhenti melakukannya kerana masalah penglihatan 5
Berhenti melakukannya kerana sebab-sebab lain atau tidak berminat untuk melakukannya 6

7. **Adakah anda menghadapi kesukaran mencari sesuatu di atas rak yang penuh dengan barang kerana penglihatan anda?**

(BACAKAN KATEGORI YANG DIPERLUKAN)

(Bulatkan Satu)

Tidak sukar langsung 1
Sedikit sukar.. 2
Sederhana sukar.. 3
Sangat sukar.. 4
Berhenti melakukannya kerana masalah penglihatan 5
Berhenti melakukannya kerana sebab-sebab lain atau tidak berminat untuk melakukannya 6

8. **Adakah anda menghadapi kesukaran membaca papan tanda jalan atau nama kedai?**
(BACAKAN KATEGORI YANG DIPERLUKAN)

(Bulatkan Satu)

Tidak sukar langsung 1
Sedikit sukar.. 2
Sederhana sukar.. 3
Sangat sukar.. 4
Berhenti melakukannya kerana masalah penglihatan 5
Berhenti melakukannya kerana sebab-sebab lain atau tidak berminat untuk melakukannya 6

9. **Disebabkan oleh penglihatan anda, adakah anda menghadapi kesukaran untuk turun anak tangga atau turun dari penghadang jalan pada keadaan cahaya malap atau pada waktu malam?**

(BACAKAN KATEGORI YANG DIPERLUKAN)

(Bulatkan Satu)

Tidak sukar langsung 1
Sedikit sukar.. 2
Sederhana sukar.. 3
Sangat sukar... 4
Berhenti melakukannya kerana masalah penglihatan 5
Berhenti melakukannya kerana sebab-sebab lain atau tidak berminat untuk melakukannya 6

10. Disebabkan oleh penglihatan anda, adakah anda menghadapi kesukaran untuk melihat objek di sisi atau tepi anda semasa berjalan?
(BACAKAN KATEGORI YANG DIPERLUKAN)

(Bulatkan Satu)

Tidak sukar langsung 1
Sedikit sukar.. 2
Sederhana sukar.. 3
Sangat sukar... 4
Berhenti melakukannya kerana masalah penglihatan 5
Berhenti melakukannya kerana sebab-sebab lain atau tidak berminat untuk melakukannya 6

11. Disebabkan oleh penglihatan anda, adakah anda menghadapi kesukaran untuk melihat reaksi orang lain terhadap apa yang anda katakan?
(BACAKAN KATEGORI YANG DIPERLUKAN)

(Bulatkan Satu)

Tidak sukar langsung 1
Sedikit sukar.. 2
Sederhana sukar.. 3
Sangat sukar... 4
Berhenti melakukannya kerana masalah penglihatan 5
Berhenti melakukannya kerana sebab-sebab lain atau tidak berminat untuk melakukannya 6

12. Disebabkan oleh penglihatan anda, adakah anda menghadapi kesukaran untuk memilih dan memadankan pakaian?
(BACAKAN KATEGORI YANG DIPERLUKAN)

(Bulatkan Satu)

Tidak sukar langsung 1
Sedikit sukar.. 2
Sederhana sukar... 3
Sangat sukar.. 4
Berhenti melakukannya kerana masalah penglihatan 5
Berhenti melakukannya kerana sebab-sebab lain atau tidak berminat untuk melakukannya 6

13. Disebabkan oleh penglihatan anda, adakah anda menghadapi kesukaran untuk pergi ke rumah seseorang, menghadiri jamuan atau pergi ke restoran?
(BACAKAN KATEGORI YANG DIPERLUKAN)

(Bulatkan Satu)

Tidak sukar langsung 1
Sedikit sukar.. 2
Sederhana sukar... 3
Sangat sukar.. 4
Berhenti melakukannya kerana masalah penglihatan 5
Berhenti melakukannya kerana sebab-sebab lain atau tidak berminat untuk melakukannya 6

14. Disebabkan oleh penglihatan anda, adakah anda menghadapi kesukaran pergi menonton wayang, persembahan atau acara sukan?
(BACAKAN KATEGORI YANG DIPERLUKAN)

(Bulatkan Satu)

Tidak sukar langsung 1
Sedikit sukar.. 2
Sederhana sukar... 3
Sangat sukar.. 4
Berhenti melakukannya kerana masalah penglihatan 5
Berhenti melakukannya kerana sebab-sebab lain atau tidak berminat untuk melakukannya 6

15. Sekarang, saya hendak tanya tentang <u>memandu kereta</u>. Pada masa sekarang adakah anda memandu kereta sekurang-kurangnya sekali-sekala?

(Bulatkan Satu)

Ya.. 1 *Langkau terus ke Soalan 15c*

Tidak 2

15a. SEKIRANYA TIDAK, TANYA: Adakah anda <u>tidak pernah</u> memandu kereta atau anda sudah <u>berhenti</u> <u>memandu kereta</u>?

(Bulatkan Satu)

Tidak pernah memandu.............1 *Langkau terus ke Bahagian 3, Soalan 17*

Sudah berhenti...........................2

15b. SEKIRANYA SUDAH BERHENTI MEMANDU: Adakah ianya disebabkan oleh masalah penglihatan atau masalah lain, atau adakah disebabkan oleh kedua-dua masalah penglihatan dan masalah lain?

(Bulatkan Satu)

Masalah penglihatan terutamanya……………… 1 *Langkau terus ke Bahagian 3, Soalan 17*

Masalah lain terutamanya………2 *Langkau terus ke Bahagian 3, Soalan 17*

Kedua-duanya iaitu penglihatan dan sebab-sebab lain…... 3 *Langkau terus ke Bahagian 3, Soalan 17*

15c. SEKIRANYA MASIH MEMANDU: Adakah anda menghadapi kesukaran memandu di tempat yang biasa anda kunjungi pada waktu siang. Bolehkah dikatakan anda menghadapi:

(Bulatkan Satu)

Tidak sukar langsung 1
Sedikit sukar......................2
Sederhana sukar...............3
Sangat sukar.....................4

16. Adakah anda menghadapi kesukaran memandu pada waktu malam? Bolehkah dikatakan anda menghadapi:
(BACAKAN KATEGORI YANG DIPERLUKAN)

(Bulatkan Satu)

Tidak sukar langsung 1
Sedikit sukar.. 2
Sederhana sukar... 3
Sangat sukar.. 4
Berhenti melakukannya kerana masalah penglihatan 5
Berhenti melakukannya kerana sebab-sebab lain atau tidak berminat untuk melakukannya 6

16a. Adakah anda menghadapi masalah memandu dalam keadaan yang sukar seperti cuaca buruk, kesesakan lalulintas, lebuhraya atau lalulintas dalam bandar. Bolehkah dikatakan anda menghadapi:
(BACAKAN KATEGORI YANG DIPERLUKAN)

(Bulatkan Satu)

Tidak sukar langsung 1
Sedikit sukar.. 2
Sederhana sukar... 3
Sangat sukar.. 4
Berhenti melakukannya kerana masalah penglihatan 5
Berhenti melakukannya kerana sebab-sebab lain atau tidak berminat untuk melakukannya 6

BAHAGIAN 3: TINDAK BALAS TERHADAP MASALAH PENGLIHATAN

Soalan berikutnya adalah mengenai bagaimana perkara-perkara yang anda lakukan mungkin terjejas kerana penglihatan anda. Bagi setiap soalan, saya mahu anda nyatakan sama ada ianya benar bagi anda untuk setiap masa, selalu, kadang-kadang, jarang-jarang, atau tidak langsung.

(Bulatkan Satu Bagi Setiap kenyataan)

**DIBACAKAN KATEGORI:**	**Setiap masa**	**Selalu**	**Kadang-kadang**	**Jarang-jarang**	**Tidak langsung**
**17. Adakah pencapaian anda kurang daripada yang anda inginkan kerana penglihatan anda?**	**1**	**2**	**3**	**4**	**5**
**18. Adakah jumlah masa bagi anda melakukan kerja atau aktiviti lain dihadkan oleh penglihatan anda?**	**1**	**2**	**3**	**4**	**5**
**19. Adakah rasa sakit atau tidak selesa pada mata atau sekeliling mata anda, contohnya, rasa panas terbakar, gatal atau sakit, menyekat anda dari melakukan apa yang ingin anda lakukan?**	**1**	**2**	**3**	**4**	**5**

Bagi setiap kenyataan berikut, tolong nyatakan sama ada ianya memang benar, selalunya benar, selalunya salah, memang salah atau anda tidak pasti.

(Bulatkan Satu Bagi Setiap kenyataan)

	Memang benar	Selalunya benar	Tidak pasti	Selalunya salah	Memang salah
20. Saya berada di rumah kebanyakan masanya kerana penglihatan saya…………………...	1	2	3	4	5
21. Saya selalu berasa kecewa kerana masalah penglihatan saya….........................	1	2	3	4	5
22. Saya mempunyai kurang kawalan ke atas perbuatan saya kerana penglihatan saya.	1	2	3	4	5
23. Disebabkan oleh penglihatan saya, saya terlalu bergantung kepada apa yang diberitahu oleh orang lain…………….	1	2	3	4	5
24. Saya memerlukan banyak bantuan dari orang lain kerana penglihatan saya.......	1	2	3	4	5
25. Saya bimbang saya akan melakukan sesuatu yang memalukan diri sendiri atau orang lain, kerana penglihatan saya. ………………….	1	2	3	4	5

Temubual ini berakhir di sini. Terima kasih kerana meluangkan masa dan membantu kami.

Lampiran Soalan-Soalan Tambahan Pilihan

SUB DOMAIN : KESIHATAN UMUM

A1. Bagaimanakah anda menilai tahap kesihatan anda secara keseluruhan, pada skala di mana kosong adalah seburuk kematian dan 10 adalah keadaan kesihatan yang terbaik mungkin?

(Bulatkan Satu)

0	1	2	3	4	5	6	7	8	9	10
**Terburuk**										**Terbaik**

SUB DOMAIN: PENGLIHATAN UMUM

A2. Bagaimanakah anda menilai penglihatan anda sekarang (dengan cermin mata atau kanta sentuh, sekiranya anda menggunakannya), pada skala dari 0 ke 10, di mana kosong bermakna keadaan penglihatan yang paling buruk, seteruk atau lebih teruk dari menjadi buta, dan 10 bermakna penglihatan yang paling baik?

(Bulatkan Satu)

0	1	2	3	4	5	6	7	8	9	10
**Terburuk**										**Terbaik**

SUB DOMAIN: PENGLIHATAN JARAK DEKAT

A3. Dengan memakai cermin mata, adakah anda menghadapi kesukaran untuk membaca tulisan kecil pada buku telefon, pada botol ubat, atau pada borang undang-undang?
Adakah anda rasa:
(BACAKAN KATEGORI YANG DIPERLUKAN)

(Bulatkan Satu)

Tidak sukar langsung 1
Sedikit sukar... 2
Sederhana sukar.. 3
Sangat sukar...4
Berhenti melakukannya kerana masalah penglihatan 5
Berhenti melakukannya kerana sebab-sebab lain atau tidak berminat untuk melakukannya 6

A4. Disebabkan oleh penglihatan anda, adakah anda menghadapi kesukaran untuk menentukan sama ada bil yang anda terima adalah tepat?
(BACAKAN KATEGORI YANG DIPERLUKAN)

(Bulatkan Satu)

Tidak sukar langsung 1
Sedikit sukar.. 2
Sederhana sukar.. 3
Sangat sukar... 4
Berhenti melakukannya kerana masalah penglihatan 5
Berhenti melakukannya kerana sebab-sebab lain atau tidak berminat untuk melakukannya 6

A5. Disebabkan oleh penglihatan anda, adakah anda menghadapi kesukaran untuk melakukan perkara-perkara seperti bercukur, mendandan rambut, atau bersolek?
(BACAKAN KATEGORI YANG DIPERLUKAN)

(Bulatkan Satu)

Tidak sukar langsung 1
Sedikit sukar.. 2
Sederhana sukar.. 3
Sangat sukar... 4
Berhenti melakukannya kerana masalah penglihatan 5
Berhenti melakukannya kerana sebab-sebab lain atau tidak berminat untuk melakukannya 6

SUB DOMAIN: PENGLIHATAN JARAK JAUH

A6. Disebabkan oleh penglihatan anda, adakah anda menghadapi masalah mengecam orang yang anda kenal di hujung sesebuah bilik yang besar?
(BACAKAN KATEGORI YANG DIPERLUKAN)

(Bulatkan Satu)

Tidak sukar langsung 1
Sedikit sukar.. 2
Sederhana sukar.. 3
Sangat sukar... 4
Berhenti melakukannya kerana masalah penglihatan 5
Berhenti melakukannya kerana sebab-sebab lain atau tidak berminat untuk melakukannya 6

A7. Disebabkan oleh penglihatan anda, adakah anda menghadapi masalah untuk mengambil bahagian di dalam sukan aktif atau aktiviti luar lain yang anda gemari (seperti golf, badminton, jogging, atau berjalan)?
(BACAKAN KATEGORI YANG DIPERLUKAN)

(Bulatkan Satu)

Tidak sukar langsung 1
Sedikit sukar.. 2
Sederhana sukar.. 3
Sangat sukar.. 4
Berhenti melakukannya kerana masalah penglihatan 5
Berhenti melakukannya kerana sebab-sebab lain atau tidak berminat untuk melakukannya 6

A8. Disebabkan oleh penglihatan anda, adakah anda menghadapi masalah untuk menonton dan menikmati rancangan di TV?
(BACAKAN KATEGORI YANG DIPERLUKAN)

(Bulatkan Satu)

Tidak sukar langsung 1
Sedikit sukar.. 2
Sederhana sukar.. 3
Sangat sukar.. 4
Berhenti melakukannya kerana masalah penglihatan 5
Berhenti melakukannya kerana sebab-sebab lain atau tidak berminat untuk melakukannya 6

SUB DOMAIN: FUNGSI SOSIAL

A9. Disebabkan oleh penglihatan anda, adakah anda menghadapi masalah melayan sahabat dan keluarga di rumah anda?
(BACAKAN KATEGORI YANG DIPERLUKAN)

(Bulatkan Satu)

Tidak sukar langsung 1
Sedikit sukar.. 2
Sederhana sukar.. 3
Sangat sukar.. 4
Berhenti melakukannya kerana masalah penglihatan 5
Berhenti melakukannya kerana sebab-sebab lain atau tidak berminat untuk melakukannya 6

SUB DOMAIN: MEMANDU

A10. [Perkara ini "memandu di dalam keadaan sukar", telah dimasukkan sebagai perkara 16a sebagai sebahagian dari set asas 25-perkara sasaran penglihatan.]

SUB DOMAIN: BATASAN PERANAN

A11. Soalan berikutnya adalah mengenai perkara yang mungkin anda lakukan disebabkan oleh penglihataan anda. Bagi setiap perkara, saya hendak anda nyatakan samaada ianya adalah benar bagi anda untuk setiap masa, selalu, kadang-kadang, jarang-jarang, atau tidak langsung.
(BACAKAN KATEGORI YANG DIPERLUKAN)

(Bulatkan Satu Bagi Setiap Baris)

	Setiap masa	Selalu	Kadang-kadang	Jarang-jarang	Tidak langsung
a. Adakah anda mendapat lebih bantuan dari orang lain kerana penglihatan anda?......................	1	2	3	4	5
b. Adakah anda terhalang untuk melakukan perkara-perkara yang boleh anda lakukan kerana penglihatan anda?	1	2	3	4	5

SUB DOMAIN: KESEJAHTERAAN/ KESUSAHAN(#A12) dan KEBERGANTUNGAN (#A13)

Soalan-soalan berikutnya adalah mengenai bagaimana anda menangani masalah penglihatan anda. Bagi setiap kenyataan berikut, tolong nyatakan sama ada ianya memang benar, selalunya benar, selalunya salah, memang salah atau anda tidak pasti

(Bulatkan Satu Bagi Setiap Baris)

	Memang benar	Selalunya benar	Tidak pasti	Selalunya salah	Memang salah
**A12. Saya cepat marah disebabkan penglihatan saya………**	**1**	**2**	**3**	**4**	**5**
**A13. Saya tidak keluar rumah bersendirian, disebabkan penglihatan saya...........**	**1**	**2**	**3**	**4**	**5**
